# A new species of *Halacarsantia* Wolff, 1989 (Crustacea, Isopoda, Asellota, Santiidae) from Wistari Reef, southern Great Barrier Reef, Australia

**DOI:** 10.3897/zookeys.173.2314

**Published:** 2012-03-02

**Authors:** Michitaka Shimomura, Niel L. Bruce

**Affiliations:** 1 Kitakyushu Museum of Natural History and Human History, Kitakyushu 805-0071 Kitakyushu, Japan; 2 Museum of Tropical Queensland, Queensland Museum and School of Marine and Tropical Biology, James Cook University, 70-102 Flinders Street, Townsville, Australia 4810; Senior Research Fellow, Department of Zoology, University of Johannesburg, Auckland Park, 2006 South Africa

**Keywords:** Isopoda, Santiidae, *Halacarsantia*, coral reefs, eastern Australia

## Abstract

*Halacarsantia acuta* **sp. n.** is described from Wistari Reef, Capricorn Group, southern Great Barrier Reef, the first record of the genus from Australia. The new species differs from its congeners in having antenna flagellum composed of 8 articles; epipod apically acute, without setae, broad maxilliped endite and pereopod 1 basis with a short projection. A key to species of the genus is provided.

## Introduction

The Santiidae is a small family of the Asellota with 28 species in five genera (Schotte et al. 2008 onwards). Species of *Halacarsantia* Wolff, 1989 are tiny (0.62–0.85 mm) and include six species, all from marine benthic habitats (Menzies and Miller 1955, Wolff 1989, Wolff and Brandt 2000, Müller 1992, Shimomura and Ariyama 2004). Two species, *Halacarsantia uniramea* (Menzies & Miller, 1955) and *Halacarsantia kussakini* Müller, 1992 has been so far described from the southern Pacific, from New Zealand and coral reefs at Mooréa (Society Islands) respectively.

The marine asellote fauna of Australia is diverse in shallow and deep waters (Kensley 1982, Wilson 1989, Merrin and Poore 2003, Just and Wilson 2004), but remains particularly poorly documented in tropical Australia, with only three named species reported from the Great Barrier Reef (Bruce 2009, Just and Wilson 2004, Kensley 1982). The genus *Halacarsantia* has not previously been recorded from Australian waters, though the genus is known from tropical and coral-reef habitats (e.g. Müller 1989, Wolff 1989, Wolff and Brandt 2000). In the present paper, we report a new species of *Halacarsantia* from the shallow coral-reef habitats of the Capricorn Group, southern Great Barrier Reef.

## Material and methods

Collections of isopods were obtained by the CReefs program organized by the Australian Institute of Marine Science (AIMS) in Heron Island in 2009. Pieces of coral rubble collected by hand during SCUBA were washed in a bucket, and isopods were extracted by decanting the suspension through a sieve with a pore size in 0.3 mm. All the specimens obtained were fixed and preserved in 95% ethanol. Each individual was dissected and prepared for observation by a light microscope (Nikon E600). The total length as indicated in “Material examined” was measured from the tip of the head to the end of the pleotelson.

The type specimens are deposited in the Museum of Tropical Queensland, Townsville (MTQ).

## Systematics

**Family Santiidae Wilson, 1987**

### Halacarsantia

Wolff, 1989

http://species-id.net/wiki/Halacarsantia

Halacarsantia Wolff, 1989: 184.

#### Type species.

*Halacarsantia justi* Wolff, 1989; by original designation.

#### Species included.

*Halacarsantia colombiensis* Wolff & Brandt, 2000, Colombia; *Halacarsantia justi* Wolff, 1989, Andaman Sea; *Halacarsantia kussakini* Müller, 1992, Mooréa; *Halacarsantia ovata* Shimomura & Ariyama, 2004, Japan; *Halacarsantia setosa* Shimomura & Ariyama, 2004, Japan; *Halacarsantia uniramea* (Menzies & Miller, 1955), New Zealand.

#### Diagnosi.

**(modified from Wolff 1989).** Body depressed, lanceolate or ovate, widest at pereonite 3, with robust setae on or near lateral margins of head, pereonites and pleotelson. Head with large, broadly rounded, frontal lobe. Eye lobes obsolete or small. No pleonite visible anterior to pleotelson. Second article of antennula with dorsolateral projection; article 5 as long as two preceding equally sized articles combined, with aesthetasc. Third article of peduncle of antenna with outer projection, with robust seta. Pereopods short, robust or slender, with robust setae, particularly on carpus. Pereopod 1 with two claws. Female operculum broader than long. Uropods uniramous.

#### Key to the species of *Halacarsantia*.

**Table:** 

1	Mandibular palp absent	2
–	Mandibular palp present	3
2	Body with many robust setae near lateral margin, without dorsal robust setae	*Halacarsantia justi*
–	Body with many robust setae on dorsum	*Halacarsantia setosa*
3	Frontal lobe of head more than 0.6 times as wide as maximum width of head; pleotelson broader than long; uropods stout	4
–	Frontal lobe of head less than 0.3 times as wide as maximum width of head; pleotelson narrower than long; uropods narrow	5
4	Coxal plates of pereonites 1, 2 and 4–7 dorsally invisible; eyes without bulging processes	*Halacarsantia ovata*
–	Coxal plates of all pereonites dorsally visible; eyes with bulging processes	*Halacarsantia colombiensis*
5	Frontal lobe of head with 17 robust setae; antenna shorter than half length of body; pereopods 5–7 broader than preceding pereopods; uropods stout	*Halacarsantia uniramea*
–	Frontal lobe of head with 6 robust setae; antenna longer than half length of body; pereopods 5–7 similar to preceding pereopods; uropods slender	6
6	Lateral margins of head each with 2 robust setae; antenna flagellum composed of 11 articles; maxilliped epipod apically blunt, with 2 setae	*Halacarsantia kussakini*
–	Lateral margins of head each with 3 robust setae; antenna flagellum composed of 8 articles; maxilliped epipod apically acute, without setae	*Halacarsantia acuta* sp. n.

### Halacarsantia acuta sp. n.

urn:lsid:zoobank.org:act:FAEAFDD4-6AE6-44BC-B733-B9274D16CF21

http://species-id.net/wiki/Halacarsantia_acuta

Figs 1 [Fig F2] 3

#### Material examined.

Holotype. ♀ (0.83 mm, with 4 eggs), Wistari Reef, Great Barrier Reef, Australia, 23 November 2009, 23°27.257’S, 151°52.840’E, northern side of reef, rubble on bommie, 3 m, stn HI09-084F, coll. N. L. Bruce & K. Schnabel (MTQ W34048).

Paratype. 1♀ (0.83 mm), same data as holotype (MTQ W31550).

#### Description of the holotype female.

**Figure 1.**
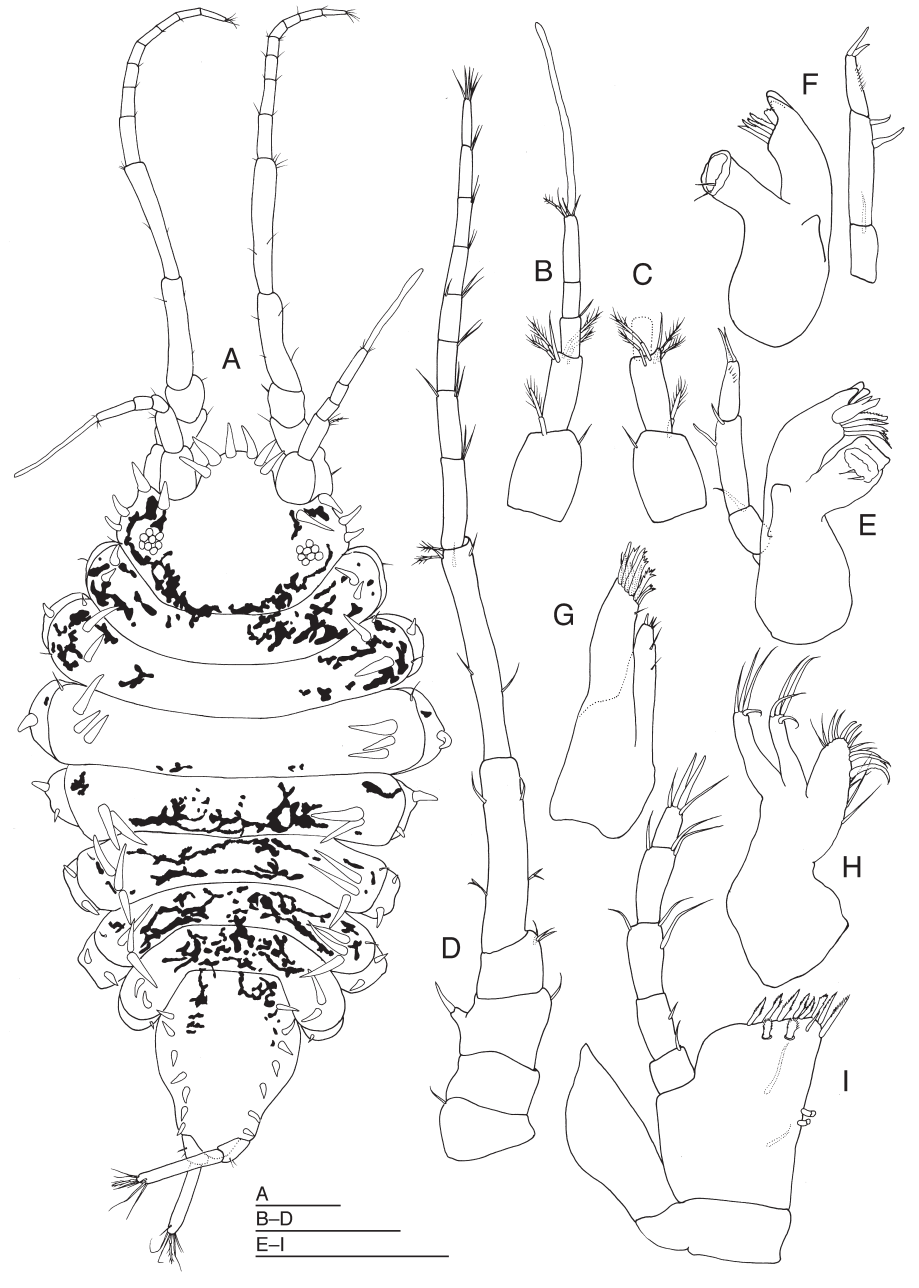
*Halacarsantia acuta* sp. n. **A–I** holotype female: **A** habitus, dorsal **B** right antennula, ventral **C** articles 1–3 of right antennula, dorsal **D** right antenna, ventral **E** left mandible, dorsal **F** right mandible, dorsal **G** right maxilla 1, ventral **H** right maxilla 2, ventral **I** left maxilliped, dorsal. Scales = 100 μm.

*Body* (Fig. 1A) 1.9 times as long as maximum width, with dark reddish brown pigment in patches. Head 1.4 times as broad as long, narrower than pereonite 1, with 1 robust seta on anterior part of eyes; frontal lobe broad and long, 0.32 times as wide as maximum width of head, with 6 long robust setae on anterior margin; lateral margins of head, each with 1 slender and 3 robust setae; posterior margin of head convex. Eyes each with 7 ommatidia. Pereonites laterally rounded; pereonites 1, 4 and 7 with 2 long robust setae near lateral margin on each side; pereonite 2 with 2 short slender setae on each side of lateral margins and 2 long robust setae near lateral margin on each side; pereonites 3, 5 and 6 with 1 short slender setae on each side of lateral margins and 3 robust setae near lateral margin on each side. Pereonites 1 to 3 increasing in length; pereonites 3 and 4 subequal in length; pereonites 5–7 subequal in length. Pereonites 1 to 3 increasing in width; pereonite 4 slightly narrower than pereonite 3; pereonites 4 to 7 decreasing in width. Coxal plates dorsally visible on all pereonites, laterally rounded; coxal plate of pereonite 1 with 1 short seta; coxal plates of pereonites 2 and 3 each with 1 robust seta; coxal plates of pereonites 4–6 each with 2 robust setae; coxal plate of pereonite 7 with 1 short seta. Pleotelson (Figs 1A, 3E, 3F) pyriform, about 1.3 times as long as wide, with 7 robust setae near dorsolateral margin on each side and 4 robust setae near ventrolateral margin on each side.

*Antennula* (Figs 1B, 1C) composed of 5 articles. Article 1 broadest, with 1 simple seta mediodistally and 1 broom seta laterodistally; article 2 slightly shorter than article 1, with 1 simple seta and 4 broom seta distally; article 3 with 1 simple seta mediodistally; article 4 as long as article 3, without setae; article 5 twice as long as article 4, with 3 simple setae, 1 broom seta and 1 aesthetasc apically.

*Antenna* (Fig. 1D): peduncle composed of 4 short stout and 2 long slender articles, and flagellum of 8 short slender articles. Article 1 with 1 simple seta laterodistally; article 2 without setae; article 3 with 1 simple seta mediodistally and 1 stout seta on lateral protrusion; article 4 with 3 simple setae mediodistally; article 5 shorter than articles 1–4 combined, with 1 simple and 1 biramous setae laterally and medially; article 6 longer and slender than article 5, with 1 lateral and 1 medial simple setae, and 3 simple and 2 broom setae; flagellar articles 1–7, each with 2 or 3 simple setae distally; flagellar article 8 with 6 simple setae apically.

*Left mandible* (Fig. 1E) palp article 1 with seta distally; article 2 longest, laterally with 2 setae; article 3 as long as article 1, with 2 apical setae and few short setae; molar process with 2 setae; *lacinia mobilis* with 2 teeth; setal row with 4 setae. Right mandible (Fig. 1F) palp article 1 with seta distally, article 2 longest, laterally with 2 setae, article 3 as long as article 1, with 2 apical setae and few short setae; incisor with 4 cusps, setal row with 4 setae; molar process stout, with 2 setae.

*Maxilla 1* (Fig. 1G) with inner lobe bearing 4 apical and 2 medial setae; outer lobe with 11 irregular and 1 simple setae distally. *Maxilla 2* (Fig. 1H) with inner lobe bearing 16 setae on margin; outer 2 lobes each with 4 apical setae.

*Maxilliped* (Fig. 1I) palp slender: article 1 shortest, with 1 seta mediodistally; article 2 about 1.9 times as long as article 1, with 1 medial seta; article 3 slightly longer than article 2, with 1 seta laterodistally and 2 setae medially; article 4 as long as article 3, with 1 seta laterodistally and 2 setae medially; article 5 narrowest, with 2 slender and 2 stout setae apically; endite broad, bearing 2 simple setae ventrally, with 8 pectinate setae distally, 1 simple and 2 fan-shaped setae on subdistal margin, and 2 coupling hooks medially; epipod lanceolate, moderately broad, with acute apex.

**Figure 2. F2:**
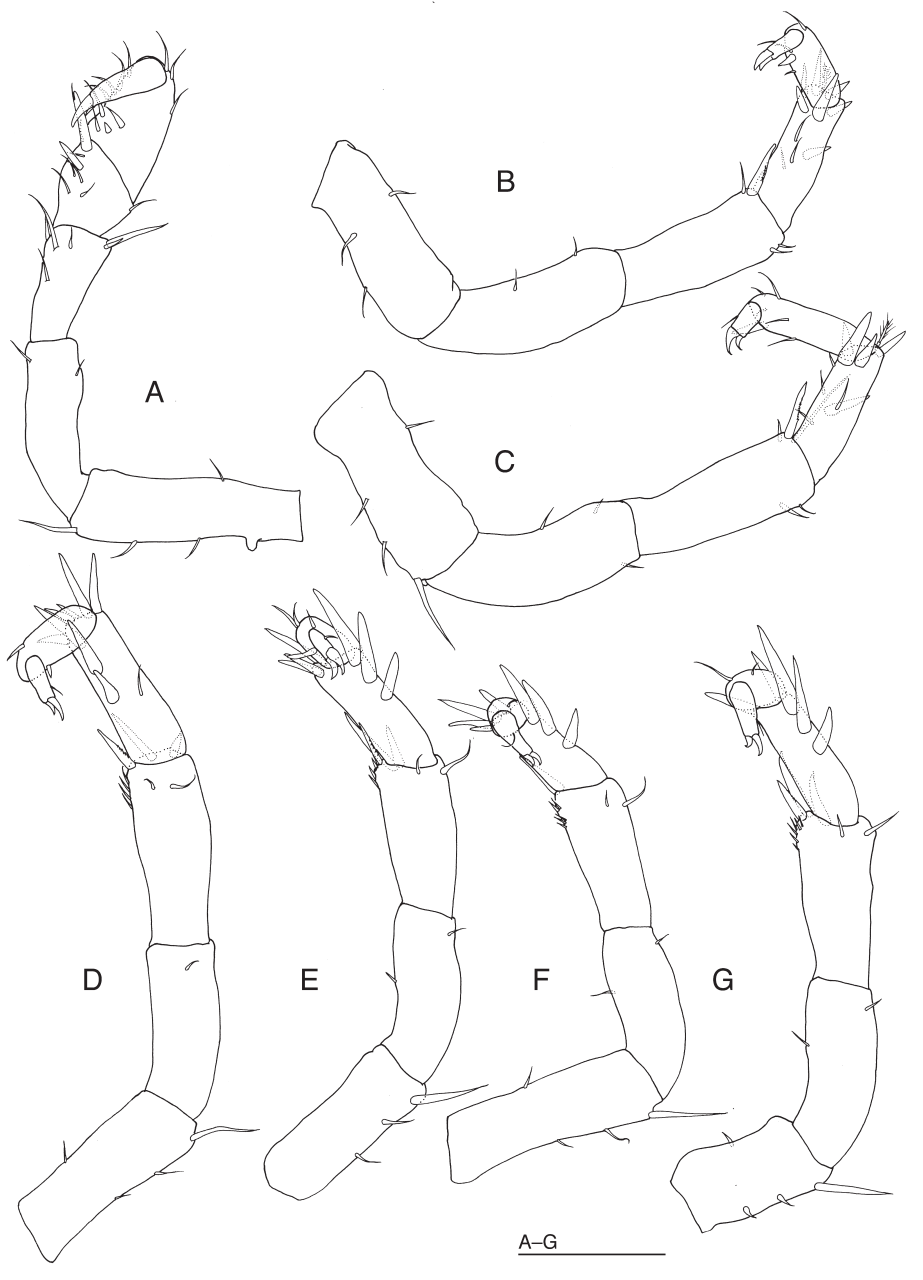
*Halacarsantia acuta* sp. n. **A–G** holotype female: **A** right pereopod 1, medial **B** right pereopod 2, dorsal **C** right pereopod 3, dorsal **D** right pereopod 4, dorsal **E** right pereopod 5, dorsal **F** right pereopod 6, dorsal **G** right pereopod 7, dorsal. Scale = 100 μm.

*Pereopod 1* (Fig. 2A): basis the longest article, with short projection ventrally, and with 3 ventral and 1 dorsal setae; ischium 0.8 as long as basis, bearing 1 ventral and 1 dorsal setae; merus trapezoidal, with 4 ventral and 2 dorsal setae; carpus trapezoidal, 0.8 as long as merus, wider than merus, ventrally with 2 stout and 6 slender setae, dorsally with 1 slender seta; propodus ovate, with 8 ventral and 3 dorsal setae; dactylus shorter than propodus, with 1 curved unguis and 1 short accessory claw.

*Pereopods 2–3* (Fig. 2B, C) subequal in shape and length; bases with 2–3 ventral and 1 dorsal setae; ischia as long as bases, with 0–1 ventral and 2 dorsal setae; meri as long as ischia, with 2 setae ventrally, 1–2 simple and 1 robust setae dorsodistally; carpi shorter than meri, with 3–4 simple, 1 broom and 5–7 robust setae; propodi shorter than carpi, with 3–5 simple and 1 robust setae; dactyli with 1 short setae, 1 curved unguis and 1 curved accessory claw. *Pereopods 4–6* (Fig. 2D–F) decreasing in length posteriorly; bases with 2 short and 1 long setae ventrally, and with 0–1 short seta dorsally; ischia with 0–1 short seta; meri with 2 short distal setae, and with 1–3 robust setae and row of short setae dorsodistally; carpi with 2–3 robust setae ventrally and 3–6 robust setae distally; propodi with 1–2 short setae and 0–1 robust seta; dactyli with 1 short seta, 1 curved unguis and 1 curved accessory claw. *Pereopod 7*: basis shorter than those of pereopods1–6, with 1 long and 2 short setae ventrally and 1 short seta dorsally; ischium with 1 dorsal and 1 ventral setae; merus with 2 short setae ventrodistally and 3 robust setae and row of spinules dorsodistally; carpus with 4 robust setae ventrally and 1 robust seta dorsodistally; propodus with 2 short setae; dactylus with 1 short seta, 1 curved unguis and 1 curved accessory claw.

**Figure 3.**
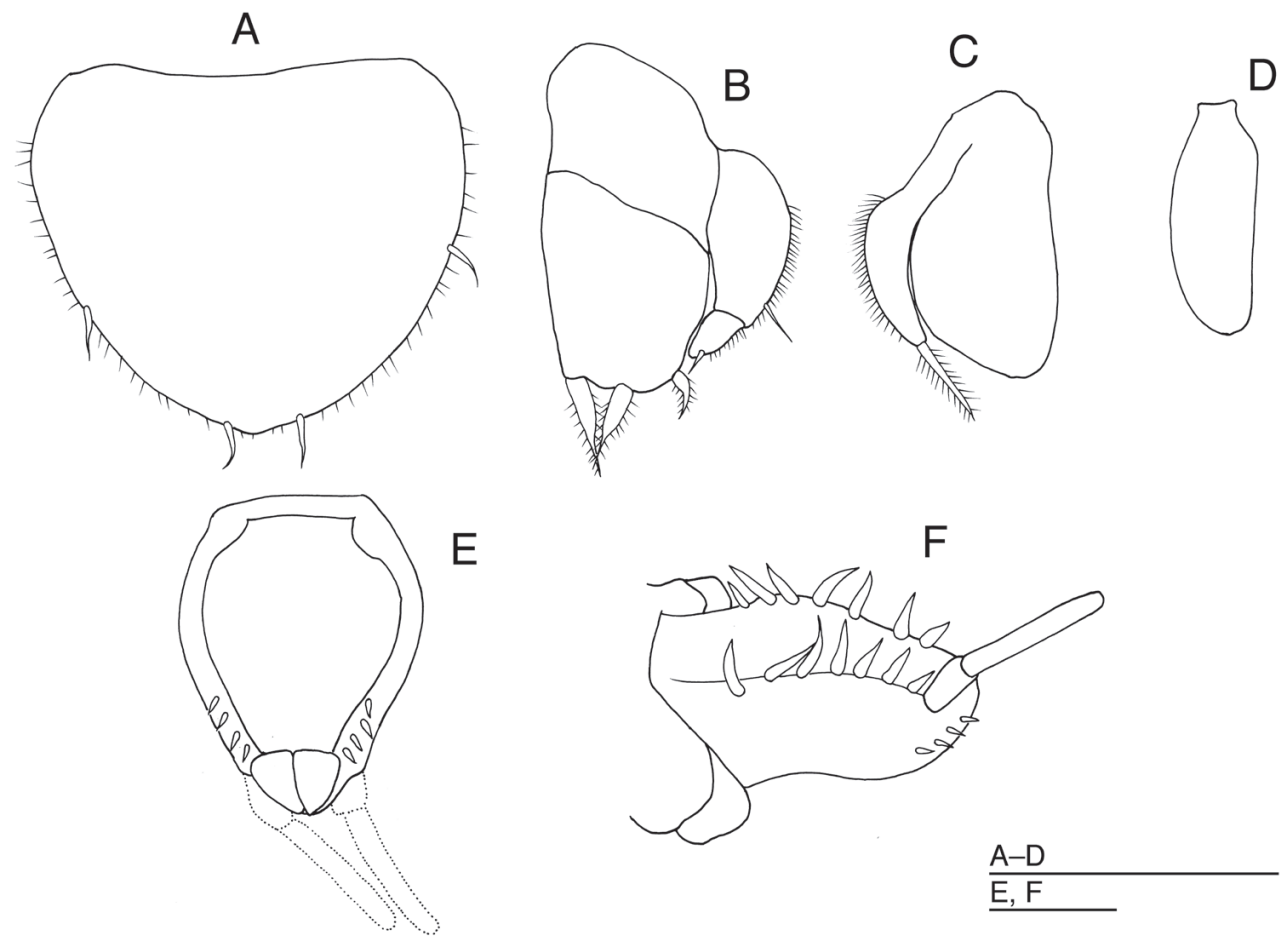
*Halacarsantia acuta* sp. n. **A–F** holotype female: **A** operculum, ventral **B** right pleopod 3, dorsal **C** right pleopod 4, ventral **D** right pleopod 5, ventral **E** pleotelson, ventral **F** pleotelson, lateral. Scales = 100 μm.

*Operculum* (Fig. 3A) 1.2 times as broad as long, with 2 lateral, 2 subapical setae, and many fine marginal setae. Pleopod 3 (Fig. 3B) with endopod bearing 3 stout, plumose setae distally; exopod narrower than endopod, bearing 1 lateral, 1 apical long simple setae, and many fine setae on convex lateral margin. Pleopod 4 (Fig. 3C) with ovate endopod; exopod uniramous, narrow, with 1 plumose seta distally, and many fine setae on lateral margin. Pleopod 5 (Fig. 3D) ovate, uniramous, about 2.8 times as long as broad, without setae.

#### Remarks.

*Halacarsantia* *acuta* sp. n. may be distinguished from its congeners in having long robust setae on the pereonites. The body shape of *Halacarsantia* *acuta* is similar *Halacarsantia kussakini* Müller, 1992 from the Society Islands, French Polynesia (type locality), but *H*. *acuta* can be separated from *Halacarsantia kussakini* by the (those of *Halacarsantia kussakini* in parentheses): 3 robust setae on each side of the head (2 setae); antenna flagellum composed of 8 articles (11 articles); maxilliped epipod apically acute, without setae (apically blunt, with 2 slender setae); maxilliped endite broad (moderately narrow); and pereopod 1 basis with a conspicuous short projection (without conspicuous projections).

#### Etymology.

The species is named after the apically acute maxilliped epipod.

## Supplementary Material

XML Treatment for Halacarsantia

XML Treatment for Halacarsantia acuta
